# Current prevalence of and obstetric outcomes in underweight Japanese women

**DOI:** 10.1371/journal.pone.0218573

**Published:** 2019-06-18

**Authors:** Shunji Suzuki

**Affiliations:** Department of Obstetrics and Gynecology, Japanese Red Cross Katsushika Maternity Hospital, Tokyo, Japan; University of Mississippi Medical Center, UNITED STATES

## Abstract

We compared the current prevalence of and obstetric outcomes in underweight Japanese women with data from 16 years ago. We reviewed the obstetric records of singleton pregnant Japanese women who delivered at our institute at ≥ 22 weeks’ gestation from 2000 through 2002 and those from 2016 through 2018. From 2000–2002 to 2016–2018, numbers of pregnant women in their twenties decreased and pregnant women in their forties increased significantly (*p* < 0.01). There were no significant changes in the prevalence of underweight pregnant women between the 2 periods. The prevalence of women whose weight gain during pregnancy was less than optimal increased (*p* = 0.049). There was no difference in obstetric outcomes between the 2 periods. Based on the current results, we cannot judge the necessity of further education to curb the increasing trend of Japanese women who desire slimness. Maternal weight may not have a high priority as an indicator of the gestational nutritional status associated with obstetric outcomes in Japan.

## Introduction

Japanese obstetricians and midwives have historically recommended limiting weight gain during pregnancy to avoid difficult deliveries; however, the trend of staying slim during pregnancy has recently been recognized as a serious problem hampering the ability to have a healthy baby [1,2]. There are many underweight women in Japan who negatively view marked weight gain during pregnancy due to their desire for slimness [3,4]. Young Japanese women's strong desire to be thin has been considered as the underlying cause [3,4]. In conjunction with these trends, the average birth weight of Japanese neonates has decreased every year. Twenty-thirty years ago, the average birth weight in Japan was about 3,300 g; however, it has now decreased to be about 3,000 g and the prevalence of low-birth-weight infants in Japan has increased to about 10% [3,4]. To date, pre-pregnancy underweightness and less than optimal weight gain have been reported to be associated with adverse obstetric outcomes such as preterm birth and fetal growth restriction [5–8]. In addition, some birth cohort studies in Japan have also been deemed suitable for epidemiological studies to demonstrate the ‘Developmental Origins of Health and Disease (DOHaD)’ indicating the developmental plasticity and mismatch concept [4,9,10]. Based on these backgrounds, the importance of nutrition and weight gain during pre-pregnancy and pregnancy has now been widely recognized in Japan [4].

Based on the hypothesis that the trend of underweight pregnant women in Japan has been increasing, in this study we compared the current prevalence of and obstetric outcomes in underweight Japanese women with those 16 years ago.

## Materials and methods

The protocol for this retrospective study was approved by the Ethics Committee of the Japanese Red Cross Katsushika Maternity Hospital. Written informed consent to analyze a retrospective database was obtained from all subjects.

We reviewed the obstetric records of singleton pregnant Japanese women who delivered at our institute at ≥ 22 weeks’ gestation from January 2000 through December 2002 and those from January 2016 through December 2018.

In this study, underweight women and their optimal weight gain during pregnancy were defined as a body mass index (BMI; kg/m^2^) during pre-pregnancy below 18.5 and 9–18 kg, respectively, according to the Institute of Medicine (IOM) and the Japanese Ministry of Health, Labour and Welfare (JMHLW) guidelines [3,11,12].

As characteristics of patients, we examined the maternal age, primiparous rate, height, body weight and BMI during pre-pregnancy, body weight at delivery, and total weight gain during pregnancy. The main obstetric outcomes were hypertensive disorders, gestational diabetes mellitus (GDM), macrosomia, low birth weight, light for gestational age (LGA), preterm premature rupture of the membranes, preterm delivery, cesarean delivery, and postpartum hemorrhage ≥ 1,000 mL according to our previous study concerning optimal gestational weight gain in Japanese women [12]. In addition, we compared the obstetric outcomes of pre-pregnancy underweight Japanese women with gestational weight gain of 9–18 kg with those showing gestational weight gain < 9 kg in 2000–2002 and 2016–2018. GDM, macrosomia, and a low birth weight were defined as described in detail previously [12]. Birth weight was classified as light for gestational age (LGA) if the weight was below the 10th percentile of the reference curves of birthweight for gestational age in Japanese singleton neonates [13]. The gestational age was calculated based on ultrasonographic findings at 9–11 weeks’ gestation in cases of spontaneous conception and embryo transfer dates when pregnancy was achieved by in vitro fertilization.

Data are expressed as the mean ± standard deviation (SD) or number (percentages). Data from 2000–2002 and 2016–2018 were compared by the Mann-Whitney U-test for continuous variables, and the *x*^2^ or Fisher’s exact test for categorical variables. Odds ratios (ORs) and 95% confidence intervals (CIs) were also calculated. Differences with *P* < 0.05 were considered significant. To examine the association between pre-pregnancy underweightness/less than optimal weight gain and obstetric outcomes, a multivariate logistic regression analysis was conducted to compare the obstetric outcomes of pre-pregnancy underweight Japanese women with optimal weight gain vs. those with gestational weight less than optimal in 2000–2002 and 2016–2018.

## Results

**Table 1** shows the prevalence of underweight Japanese women in each age group for 2000–2002 and 2016–2018. Pregnant women in their twenties decreased and pregnant women in their forties increased significantly (*p* < 0.01) from 2000–2002 to 2016–2018. The prevalence of underweight pregnant women increased in all ages; however, there were no significant changes in the prevalence. For example, the *p*-value of the difference in those in their forties between the 2 study periods was 0.30. Compared with pregnant women in their teens, twenties, and thirties, the prevalence of underweight women was lower by those in their forties (vs. teens: Crude OR: 0.429, 95% CI: 0.28–0.66, *p* < 0.01; vs. twenties: Crude OR: 0.512, 95% CI: 0.40–0.66, *p* < 0.01; vs. thirties: Crude OR: 0.634, 95% CI: 0.49–0.82, *p* < 0.01). The prevalence of underweight women in their twenties decreased and women in their forties increased significantly (p < 0.01) from 2000–2002 to 2016–2018.

**Table 1:** Prevalence of underweight Japanese women in each age group in 2000–2002 and 2016–2018.

Years	2000–2002			2016–2018			*P*-value^c^
	Total	Underweight women	Prevalence of underweight women^a^	Total	Underweight women	Prevalence of underweight women^a^	
Maternal age (years old)							
< 20	105 (1.7)	20 (19.0)	2.2	80 (1.7)	17 (21.3)	2.4	0.87
20–29	2,053 (32.80)	349 (17.0)	37.5	1,255 (27.0)^b^	224 (17.8)	31.2	< 0.01
30–39	3,843 (61.5)	541 (14.1)	58.2	2,826 (60.7)	425 (15.0)	59.3	0.69
≥ 40	252 (4.0)	20 (7.9)	2.2	491 (10.6)^b^	52 (10.6)	7.3	< 0.01
Total	6253 (100)	930 (14.9)	100	4,652 (100)	717 (15.4)	100	

^a^Values are numbers (percentages) or percentage.

^b^*P* < 0.01 vs. 2000–2003.

^c^*P*-value of prevalence of underweight women in 2000–2002 vs. 2016–2018.

**Table 2** shows the clinical characteristics of underweight Japanese women except for the maternal age in 2000–2002 and 2016–2018. The prevalence of primiparous women increased significantly (OR: 1.42, 95% CI: 1.2–1.7, *p* < 0.01); however, there were no significant differences in the other variables such as maternal BMI or gestational weight gain.

**Table 2:** Clinical characteristics of underweight Japanese women except for maternal age in 2000–2002 and 2016–2018.

	2000–2002	2016–2018	*P*-value
	930	717	
Primiparous women	473 (50.9)	445 (62.4)	< 0.01
Maternal height (cm)	159±5.4	159±5.9	0.46
Maternal weight at pre-pregnacy (kg)	43.5±3.3	43.4±3.8	0.41
Maternal body mass index at pre-pregnancy	17.2±0.7	17.2±0.8	0.51
Gestational weight gain (kg)	11.9±3.0	11.3±2.8	0.24
Maternal weight at delivery (kg)	55.4±3.8	54.7±4.2	0.12

Values are numbers (percentages) or numbers ± standard deviation.

**Table 3** shows the obstetric outcomes of underweight Japanese women in 2000–2002 and 2016–2018. The prevalence of pregnant women whose weight gain was less than optimal increased (*p* = 0.049), while the prevalence of those who achieved optimal weight gain decreased (*p* = 0.046). There was no difference in obstetric outcomes between underweight Japanese women in 2000–2002 and 2016–2018.

**Table 3:** Obstetric outcomes of underweight Japanese women in 2000–2002 and 2016–2018.

Years	2000–2002	2016–2018	*P*-value
	930	717	
Weight gain during pregnancy			
< 9 kg	166 (17.8)	155 (21.6)	0.06
9–18 kg	762 (81.9)	559 (77.9)	0.046
> 18 kg	2 (0.2)	3 (0.4)	0.66
Maternal weight at delivery	55.4±3.8	54.7±4.2	0.12
Hypertensive disorders	31 (3.3)	28 (3.9)	0.59
Gestational diabetes melitus	12 (1.3)	11 (1.5)	0.42
Preterm premature rupture of the membranes	28 (3.0)	23 (3.2)	0.89
Preterm delivery	83 (9.0)	69 (9.6)	0.67
Cesarean delivery	131 (14.1)	120 (16.7)	0.15
Neonatal birth weight			
< 2,500 g	130 (14.0)	123 (17.2)	0.07
≥ 4,000 g	4 (0.4)	4 (0.6)	0.73
Light for gestational age infants	115 (12.4)	86 (12.0)	0.88
Postpartum hemorrhage ≥ 1,000 mL	75 (8.1)	70 (9.8)	0.25

Values are numbers (percentages) or numbers ± standard deviation.

**Table 4** shows the obstetric outcomes of pre-pregnancy underweight Japanese women whose weight gain was less than optimal and those who achieved optimal weight gain in the both periods. There was no difference in these obstetric outcomes between underweight Japanese women in 2000–2002 and 2016–2018 regardless of the weight gain level.

**Table 4:** Obstetric outcomes of pre-pregnancy underweight Japanese women with gestational weight gain < 9 kg in 2000–2002 and 2016–2018.

Years	2000–2002	2016–2018	*P*-value
Weight gain less than optimal	166	155	
Hypertensive disorders	7 (4.2)	8 (5.2)	0.61
Gestational diabetes melitus	1 (0.6)	0 (0)	1.00
Preterm premature rupture of the membranes	6 (3.6)	4 (2.6)	0.75
Preterm delivery	15 (9.0)	12 (7.7)	0.67
Cesarean delivery	24 (14.5)	25 (16.1)	0.94
Neonatal birth weight			
< 2,500 g	42 (25.3)	41 (26.4)	0.55
≥ 4,000 g	0 (0)	0 (0)	1.00
Light for gestational age infants	38 (22.9)	31 (20.0)	0.59
Postpartum hemorrhage ≥ 1,000 mL	11 (6.6)	10 (6.5)	0.95
Optimal weight gain	762	559	
Hypertensive disorders	24 (3.1)	19 (3.4)	0.80
Gestational diabetes melitus	11 (1.4)	11 (2.0)	0.46
Preterm premature rupture of the membranes	22 (2.9)	19 (3.4)	0.60
Preterm delivery	68 (9.0)	57 (10.2)	0.44
Cesarean delivery	10.5 (13.7)	95 (17.0)	0.11
Neonatal birth weight			
< 2,500 g	88 (11.5)	82 (14.7)	0.09
≥ 4,000 g	2 (0.3)	2 (0.4)	0.76
Light for gestational age infants	77 (10.1)	55 (9.6)	0.87
Postpartum hemorrhage ≥ 1000 mL	62 (8.1)	59 (10.6)	0.13

Values are numbers (percentages) or numbers ± standard deviation.

In both periods of 2000–2002 and 2016–2018, optimal weight gain was associated with a decreased incidence of neonatal birth weight < 2,500 g (2000–2002: crude OR 0.385, 95% CI 0.26–0.58, *p* < 0.01; 2016–2018: crude OR 0.478, 95% CI 0.31–0.73, *p* < 0.01) and light for gestational weight infants (2000–2002: crude OR 0.379, 95% CI 0.25–0.58, *p* < 0.01; 2016–2018: crude OR 0.437, 95% CI 0.27–0.71, *p* < 0.01) as depicted in the supplemental table. There were no significant differences in the other obstetric outcomes of pre-pregnancy underweight women with gestational weight gain of 9–18 kg and those with gestational weight gain < 9 kg. **Table 5** shows the results of multivariate analysis for obstetric outcomes of pre-pregnancy underweight Japanese women with gestational weight gain of 9–18 kg compared with those with gestational weight gain < 9 kg in 2000–2002 and 2016–2018. Using a multivariate analysis, optimal weight gain was independently associated with the decreased incidence of adverse obstetric outcomes such as neonatal birth weight < 2,500 g and light for gestational weight infants.

**Table 5:** Results of multivariate analysis for obstetric outcomes of pre-pregnancy underweight Japanese women with gestational weight gain of 9–18 kg compared with those with gestational weight gain < 9 kg in 2000–2002 and 2016–2018.

Obstetric outcomes	Gestational weight gain	Adjusted odds ratio	95% confidence interval	*P*-value
Neonatal birth weight < 2,500 g				
2000–2002	< 9 kg	1		
	9–18 kg	0.495	0.35–0.60	< 0.01
2016–2018	< 9 kg	1		
	9–18 kg	0.578	0.40–0.86	< 0.01
Light for gestational age infants				
2000–2002	< 9 kg	1		
	9–18 kg	0.611	0.41–0.89	< 0.01
2016–2018	< 9 kg	1		
	9–18 kg	0.598	0.38–0.90	< 0.01

## Discussion

Based on recent studies in Japan [2,5,6,12,14], it may be necessary for Japanese women to view weight gain during pregnancy more positively. In addition, the current results may support some previous studies indicating the association between pre-pregnancy underweightness/ less than optimal gestational weight gain and adverse obstetric outcomes in any period [5–8]. However, in this study, the prevalence or obstetric outcomes of Japanese underweight women did not change over the 16-year period. The prevalence of underweight pregnant women increased in all ages, but it was non-significant.

To date, optimal weight gain during pregnancy for Japanese women to promote favorable perinatal outcomes has been explored, although this may not be the sole contributing factor [2,5,6,12,14]. The accumulation of evidence showing that the body composition of Asians differs from that of other races has led to concerns over whether a single set of guidelines applied globally is appropriate [15,16], although the IOM guidelines are the most widely used guidelines on gestational weight gain [1]. Reduced weight gain has not been observed to lower the risk of cesarean delivery or lead to faster postpartum weight reduction [2]; however, some slender pregnant women still desire to strictly control their weight in Japan. We had predicted that young women are the main persons desiring slimness [3,4]; however, the present results revealed no differences in the trend toward desiring slimness among the age groups, with this trend increasing at all ages.

Over the past 16 years, the number of pregnant women among older age groups has increased significantly. Advances in assisted reproductive technology have led to an increase in elderly primiparous pregnant women in Japan. An advanced maternal age has been associated with adverse outcomes, especially maternal complications including cesarean section and preeclampsia [17,18]. The obstetric outcomes associated with maternal age have also been recognized to differ by conception method and parity. If pre-pregnancy underweightness increases the occurrence of adverse obstetric outcomes, the change in the maternal age led us to expect a change in the incidence of adverse obstetric outcomes in underweight women; however, in this study the obstetric outcomes in underweight women did not change. This may indicate the progress of perinatal care in Japan. Otherwise, maternal weight may not have a high priority as an indicator of the gestational nutritional status associated with obstetric outcomes in Japan. Based on the current results, we cannot judge the necessity of further education to curb the increasing trend of Japanese women who desire slimness. Indicators other than maternal weight may be sought to promote favorable perinatal outcomes.

In this study, a shift in the age of underweight Japanese women to 40 was observed. Considering the current result of obstetric outcomes, the characteristics of underweightness in young women may differ from those in older women. Because infertility treatment is expensive in Japan, older pregnant Japanese women who have received such treatment may be financially secure. Such women tend to be more health conscious and their diet shows a good nutritional balance to avoid obesity [19–21]. Therefore, the underweightness of older women may be a more significant indicator of health than that of young women.

There were some limitations of this study. Firstly, our institute is located in downtown Tokyo, Japan. There are differences in clinical backgrounds among pregnant women living in various areas of Tokyo, Japan [19]. Therefore, the current observations may not necessarily represent the trend of all women in Japan. Secondly, the sample size may have been too small to examine the influence of age on primiparous pregnancy because the number of underweight women aged 40 years or over in 2000–2002 was only 20. In addition, optimal weight gain during pregnancy for Japanese women has not been well-examined [2,5,6,12,14]. To promote favorable perinatal outcomes, we have been exploring optimal weight gain during pregnancy, although what really matters may not be solely indicated by the range of body weight [19]. Therefore, we remain unsure whether the criterion in this study is actually suitable for Japanese women.

In conclusion, the prevalence or obstetric outcomes of underweight Japanese women did not change during the 16-year period of this study. A further large-scale study may be needed.

## Supporting information

S1 Table Comparison of obstetric outcomes of pre-pregnancy underweight Japanese women with gestational weight gain of 9–18 kg vs. those with gestational weight gain < 9 kg in 2000–2002 and 2016–2018. (DOC)

## References

[1] Normile D: Staying slim during pregnancy carries a price. Science. 2018; 361(6401): 440 10.1126/science.361.6401.440 30072522

[2] Morisaki N, Nagata C, Jwa SC, Sago H, Saito S, Oken E, et al: Pre-pregnancy BMI-specific optimal gestational weight gain for women in Japan. J Epidemiol. 2017 10;27(10):492–498. 10.1016/j.je.2016.09.013 28579106 PMC5602799

[3] Promotion Council for Healthy Parents and Children 21 (second edition) (in Japanese). Ministry of Health, Labour and Welfare, 2015. http://rhino3.med.yamanashi.ac.jp/sukoyaka2/english.html (June 15, 2016)

[4] Sata F: Developmental Origins of Health and Disease (DOHaD) and Epidemiology (in Japanese). Nihon Eiseigaku Zasshi. 2016; 71: 41–46. 10.1265/jjh.71.41 26832616

[5] Nomura K, Kido M, Tanabe A, Nagashima K, Takenoshita S, Ando K: Investigation of optimal weight gain during pregnancy for Japanese Women. Sci Rep. 2017; 7: 2569 10.1038/s41598-017-02863-1 28566718 PMC5451426

[6] Enomoto K, Aoki S, Toma R, Fujiwara K, Sakamaki K, Hirahara F: Pregnancy outcomes based on pre-pregnancy body mass index in Japanese women. PLoS One. 2016; 11: e0157081 10.1371/journal.pone.0157081 27280958 PMC4900523

[7] Triunfo S, Lanzone A: Impact of maternal under nutrition on obstetric outcomes. J Endocrinol Invest. 2015; 38: 31–38. 10.1007/s40618-014-0168-4 25194427

[8] Papazian T, Abi Tayeh G, Sibai D, Hout H, Melki I, Rabbaa Khabbaz L: Impact of maternal body mass index and gestational weight gain on neonatal outcomes among healthy Middle-Eastern females. PLoS One. 2017; 12(7): e0181255. 10.1371/journal.pone.0181255 PMC5513447 28715482

[9] Gluckman PD, Hanson MA, Beedle AS: Early life events and their consequences for later disease: a life history and evolutionary perspective. Am J Hum Biol. 2007; 19: 1–19. 10.1002/ajhb.20590 17160980

[10] Gillman MW, Barker D, Bier D, Cagampang F, Challis J, Fall C, et al: Meeting report on the 3rd International Congress on Developmental Origins of Health and Disease (DOHaD). Pediatr Res. 2007; 61: 625–629. 10.1203/pdr.0b013e3180459fcd 17413866

[11] Rasmussen KM, Yaktine AL, Eds: Weight gain during pregnancy: reexamining the guidelines Washington, DC: National Academies Press; 2009. 20669500

[12] Suzuki S: Optimal weight gain during pregnancy in Japanese women. J Clin Med Res. 2016; 8: 787–792. 10.14740/jocmr2723w 27738479 PMC5047016

[13] Ogawa Y, Iwamura T, Kuriya N, Nishida H, Takeuchi H, Takada M, et al: Birth size standards by gestational age for Japanese neonates (in Japanese). Acta Neonatol Jpn. 1998; 34: 624–632.

[14] Suzuki S: Gestational weight gain in Japanese Women with favorable perinatal outcomes. J Clin Med Res. 2017; 9: 64–66. 10.14740/jocmr2810w 27924177 PMC5127217

[15] Suzuki S: Optimal pre-pregnancy body mass index cut-offs for obesity in Japan. J Clin Med Res. 2017; 9: 180–181. 10.14740/jocmr2883w 28090236 PMC5215024

[16] WHO Expert Consultation: Appropriate body-mass index for Asian populations and its implications for policy and intervention strategies. Lancet. 2004; 363: 157–163. 10.1016/S0140-6736(03)15268-3 14726171

[17] Ogawa K, Urayama KY, Tanigaki S, Sago H, Sato S, Saito S, et al: Association between very advanced maternal age and adverse pregnancy outcomes: a cross sectional Japanese study. BMC Pregnancy Childbirth. 2017; 17(1): 349 10.1186/s12884-017-1540-0 29017467 PMC5635576

[18] Wennberg AL, Opdahl S, Bergh C, Aaris Henningsen AK, Gissler M, Romundstad LB, et al:Effect of maternal age on maternal and neonatal outcomes after assisted reproductive technology. Fertil Steril. 2016; 106: 1142–1149.e14. 10.1016/j.fertnstert.2016.06.021 27399261

[19] Suzuki S: Optimal weight gain during pregnancy in Japanese women: is it Okay? J Clin Med Res. 2018; 10: 279–280. 10.14740/jocmr3348w 29416590 PMC5798278

[20] Yoshiike N, Koyama T, Miyoshi M: Epidemiological aspects of underweight in women–Japan and overseas- (in Japanese). Obes Res. 2018; 24: 16–21.

[21] Suzuki S: Health guidance during pregnancy (in Japanese). J Jpn Soc Perin Neon Med 2018: 54: 434.

