# Supplementary material for: Current prevalence of and obstetric outcomes in underweight Japanese women
Source: PLoS One. 2019 Jun 18;14(6):e0218573. doi: 10.1371/journal.pone.0218573 (PMC6581261; doi:10.1371/journal.pone.0218573)
Supplement: S1 Table — (DOC) [file pone.0218573.s001.doc]

|  |  | | |  | | |  |
| --- | --- | --- | --- | --- | --- | --- | --- |
|  |  |  |  |  |  |  |  |
|  |  |  |  |  |  |  |  |
|  |  |  |  |  |  |  |  |
|  |  |  |  |  |  |  |  |
|  |  |  |  |  |  |  |  |
|  |  |  |  |  |  |  |  |
|  |  |  |  |  |  |  |  |

|  |  |  |  |
| --- | --- | --- | --- |
|  |  |  |  |
|  |  |  |  |
|  |  |  |  |
|  |  |  |  |
|  |  |  |  |
|  |  |  |  |
|  |  |  |  |

|  |  |  |  |
| --- | --- | --- | --- |
|  |  |  |  |
|  |  |  |  |
|  |  |  |  |
|  |  |  |  |
|  |  |  |  |
|  |  |  |  |
|  |  |  |  |
|  |  |  |  |
|  |  |  |  |
|  |  |  |  |
|  |  |  |  |
|  |  |  |  |
|  |  |  |  |
|  |  |  |  |
|  |  |  |  |
|  |  |  |  |

|  |  |  |  |
| --- | --- | --- | --- |
|  |  |  |  |
|  |  |  |  |
|  |  |  |  |
|  |  |  |  |
|  |  |  |  |
|  |  |  |  |
|  |  |  |  |
|  |  |  |  |
|  |  |  |  |
|  |  |  |  |
|  |  |  |  |
|  |  |  |  |
|  |  |  |  |
|  |  |  |  |
|  |  |  |  |
|  |  |  |  |
|  |  |  |  |
|  |  |  |  |
|  |  |  |  |
|  |  |  |  |
|  |  |  |  |
|  |  |  |  |

|  |  |  |  |  |
| --- | --- | --- | --- | --- |
|  |  |  |  |  |
|  |  |  |  |  |
|  |  |  |  |  |
|  |  |  |  |  |
|  |  |  |  |  |
|  |  |  |  |  |
|  |  |  |  |  |
|  |  |  |  |  |
|  |  |  |  |  |
|  |  |  |  |  |

**S1 Table.** Comparison of obstetric outcomes of pre-pregnancy underweight Japanese women with gestational weight gain of 9-18 kg vs. those with gestational weight gain < 9 kg in 2000-2002 and 2016-2018.

|  | 2000-2002 | | | 2016-2018 | | |
| --- | --- | --- | --- | --- | --- | --- |
|  | *P*-value | Odds ratio | 95% CI | *P*-value | Odds ratio | 95% CI |
| Hypertensive disorders | 0.48 | 0.739 | 0.32-1.7 | 0.31 | 0.641 | 0.28-1.5 |
| Gestational diabetes melitus | 0.70 | 2.42 | 0.40-15 | 0.08 | Inf. | 0.81-Inf. |
| Preterm premature rupture of the membarnes | 0.62 | 0.793 | 0.33-1.9 | 0.61 | 1.33 | 0.47-3.8 |
| Preterm de,ivery | 0.98 | 0.986 | 0.55-1.8 | 0.44 | 1.35 | 0.71-2.6 |
| Cesarean delivery | 0.81 | 0.946 | 0.59-1.8 | 0.80 | 1.07 | 0.66-1.7 |
| Neonatal birth weight |  |  |  |  |  |  |
| < 2500g | < 0.01 | 0.385 | 0.26-0.58 | < 0.01 | 0.474 | 0.31-0.73 |
| ≥ 4000g | 0.51 | Inf. | 0.11-Inf. | 0.46 | Inf. | 0.14-Inf. |
| Light for gestational age infants | < 0.01 | 0.379 | 0.25-0.58 | < 0.01 | 0.437 | 0.27-0.71 |
| Postpartum hemorrhage ≥ 1000 ml | 0.63 | 1.25 | 0.65-2.4 | 0.13 | 1.71 | 0.86-3.4 |

CI, confidence interval

Inf., infinity
